# Clinical practice of cangrelor use in Austrian ACS patients: a real-world multicenter registry

**DOI:** 10.3389/fcvm.2026.1857088

**Published:** 2026-08-18

**Authors:** Christoph Beyer, Matthias Frick, Gabor Toth-Gayor, Hannes Alber, Jolanta Siller-Matula, Günter Christ, Gerald Zenker, Kurt Huber, Raphael Wilfing, Herwig Schuchlenz, Fabian Plank, Guy Friedrich

**Affiliations:** 1 Department of Cardiology, Medical University Hospital Innsbruck, Innsbruck, Austria; 2 Department of Cardiology, Hospital of Feldkirch, Feldkirch, Austria; 3 Department of Cardiology, Medical University Hospital Graz, Graz, Austria; 4 Department of Cardiology, Hospital of Klagenfurt, Klagenfurt, Austria; 5 Department of Cardiology, Medical University Vienna, Vienna, Austria; 6 Department of Cardiology, Clinic Favoriten, Vienna Healthcare Group, Vienna, Austria; 7 Department of Cardiology, Hospital of Hochsteiermark, Hochsteiermark, Austria; 8 Austrian Heart Foundation, Vienna, Austria; 9 Department of Cardiology, Hospital of Graz West, Graz, Austria

**Keywords:** acute coronary syndrome, cangrelor, dual antiplatelet therapy, P2Y_12_ inhibitors, percutaneous coronary intervention

## Abstract

**Introduction:**

Cangrelor is the only available intravenous P2Y_12_ inhibitor and is primarily used in patients with acute coronary syndromes (ACS), especially those presenting with cardiac arrest without prior oral P2Y_12_ therapy. Despite guideline recommendations and evidence from the CHAMPION trials, real-world data on its use remain limited. This study evaluated real-world cangrelor use in patients with ACS, with a particular focus on transition strategies from oral P2Y₁₂ inhibitor therapy and associated treatment patterns.

**Methods:**

We performed a retrospective cross-sectional analysis using data from a nationwide Austrian registry including 10 high-volume percutaneous coronary intervention (PCI) centers over a 7-year period. The timing of concomitant antiplatelet and anticoagulant therapy was assessed. Major adverse cardiovascular events (MACE) and bleeding complications occurring during the index hospitalization, for up to 7 days, were recorded.

**Results:**

A total of 626 patients were consecutively enrolled over a 7-year period. Cangrelor was administered for a median duration of 2.5 h. Marked inter-center differences were observed in transition strategies and pre-procedural antithrombotic therapy. Overall, 69% of patients received aspirin and heparin alone, whereas 31% also received an oral P2Y_12_ inhibitor before cangrelor initiation. Among patients transitioned to a thienopyridine, 9 (6%) received clopidogrel and 6 (4%) received prasugrel before cangrelor discontinuation. Major bleeding (BARC ≥3) occurred in 46 patients (7%), without observed differences according to the timing of oral P2Y_12_ inhibitor administration.

**Conclusion:**

This nationwide real-world study highlights considerable variability in the clinical use of Cangrelor and its integration with oral P2Y_12_ inhibitors.

## 1 Introduction

Ischemic heart disease remains the leading cause of death globally, with acute coronary syndrome (ACS) frequently serving as its initial clinical presentation (1, 2). The rupture of atherosclerotic plaques and the subsequent release of thrombogenic material are central to the pathophysiology of ACS, triggering platelet aggregation and thrombus formation (3, 4). Treating the resulting coronary obstruction with percutaneous coronary intervention (PCI) further enhances platelet activation (5). Consequently, effective pharmacological platelet inhibition—particularly through P2Y_12_ receptor antagonists—is crucial in ACS patients undergoing PCI to prevent ischemic complications (2).

Beyond oral P2Y_12_ inhibitors, cangrelor offers an intravenous alternative for ACS patients who are unsuitable for oral antiplatelet therapy, helping to reduce peri- and post-PCI complications (2, 6). However, real-world evidence regarding its clinical use remains limited, prompting the establishment of the Austrian Registry for Cangrelor Application (ARECA) (7).

Although current recommendations restrict cangrelor use to P2Y_12_ -inhibitor–naïve patients, emerging real-world data suggest that a notable proportion of individuals with ST-elevation myocardial infarction (STEMI) receive oral P2Y_12_ inhibitors prior to periprocedural cangrelor administration. However, there is currently no dedicated analysis addressing this scenario (2, 8).

The aim of our nationwide analysis was to evaluate the utilization patterns and clinical outcomes of cangrelor in ACS patients with different platelet inhibition strategies.

## 2 Methods and materials

### 2.1 Study design

This retrospective cohort study was based on data from the nationwide ARECA registry, which included contributions from ten tertiary care hospitals and high-volume PCI centers.

The registry was approved by the respective local ethics committees of all participating institutions prior to its initiation. Due to its retrospective design and the use of anonymized data, individual informed consent from patients was not required. The study was conducted in accordance with the principles of the Declaration of Helsinki and complied with European data protection regulations.

### 2.2 Study population

This registry included all consecutive patients presenting with ACS who underwent PCI between 2015 and 2022 and received peri-procedural intravenous cangrelor, administered as a 30 µg/kg body weight bolus followed by a continuous infusion of 4 µg/kg/min, in accordance with current guidelines (2).

For this retrospective cohort study, only patients from the Austrian Registry for Cangrelor Application (ARECA) were included.

### 2.3 Statistical analysis

Continuous variables were tested for normal distribution and are expressed as means ± standard deviation, or median (IQR) in case of skewed data. Categorical data are given as absolute values and percentages and tested using Pearson's Chi-square or Fisheŕs exact test as appropriate. The Wilcoxon test or the Mann–Whitney U test was used to compare data groups. A *p*-value of less than 0.05 was considered statistically significant.

As the analyses were exploratory and descriptive, no adjustment for multiple testing was performed, and *p*-values are presented descriptively and should not be interpreted as evidence for confirmatory hypothesis testing.

All statistical analysis were conducted using and R (V4.5.0 R foundation, Vienna, Austria).

### 2.4 Outcomes

The primary objective of this study was to characterize cangrelor administration patterns and evaluate potential differences in its clinical use. Particular emphasis was placed on the timing of the first oral P2Y_12_ inhibitor administration in relation to the index percutaneous coronary intervention (PCI), categorized as before, during, or after the procedure. In addition, we assessed clinical outcomes during the index hospital period, including major bleeding and major adverse cardiovascular events (MACE). Major bleeding was defined as Bleeding Academic Research Consortium (BARC) type ≥3 (9). MACE was defined as a composite of cardiovascular death, stroke, recurrent myocardial infarction, refractory angina, and repeat coronary revascularization, in accordance with previous observational studies and the Fourth Universal Definition of Myocardial Infarction (10, 11).

Follow-up was limited to the in-hospital period, with a maximum duration of 7 days following the index PCI. A centralized core laboratory served as a clinical event adjudication committee. It reviewed and harmonized all reported outcomes.

## 3 Results

### 3.1 Baseline characteristics

The final analysis included 626 consecutive patients with acute coronary syndrome (ACS) who received periprocedural cangrelor during percutaneous coronary intervention (PCI) between 2015 and 2022 at ten major tertiary care centers in Austria. Of these, 424 patients (68%) presented with ST-segment elevation myocardial infarction (STEMI) and 202 (32%) with non-ST-segment elevation myocardial infarction (NSTEMI). Overall, 149 patients (24%) were female. The median age was 61 years, and the mean body mass index (BMI) was 27 kg/m^2^. Prior to PCI, 252 patients (40%) were intubated, while 356 (57%) received opioid analgesia, most commonly morphine (34%, *n* = 214) or fentanyl (24%, *n* = 149), with piritramide administered in only one patient (<1%).

A history of coronary artery disease was present in 51 patients (11%), including 36 (8%) with prior coronary stent implantation and 15 (3%) with previous and/or concomitant coronary artery bypass graft (CABG) surgery. Moderate renal impairment [estimated glomerular filtration rate (eGFR) < 60 mL/min/1.73 m^2^] was present in 279 patients (45%), while 28 patients (4%) had severe renal impairment (eGFR <30 mL/min/1.73 m^2^).

Baseline characteristics are summarized in Table 1.

**Table 1:** Demographics and baseline characteristics.

	Total	
Items	n	m
Sex	626	
female		149 (24%)
male		477 (76%)
Age (median)	626	61 (IQR 17)
Weight [kg]	512	101 ± 40
Height [cm]	432	170 ± 26
BMI [kg/m²]	421	27 ± 4
Arterial hypertension	522	342 (66%)
Smoking	521	243 (47%)
Diabetes mellitus	520	123 (24%)
Hypercholesterolaemia	521	282 (54%)
Positive family history	486	109 (22%)
Chronic kidney disease	470	
GFR <60		279 (59%)
GFR <45		69 (15%)
GFR <30		28 (6%)
Previous coronary artery disease	464	
Stents		36 (8%)
Bypass		15 (3%)
Myocardial Infarction	626	
NSTEMI		202 (32%)
STEMI		424 (68%)
Intubation	626	252 (40%)
Multivessel	626	152 (24%)
Left Main	626	36 (6%)
Number of Stents (median)	526	1 (IQR 1)
Thrombus Aspiration	626	104 (17%)

### 3.2 Variations in antiplatelet timing across Austrian centers

**Figure 1:**
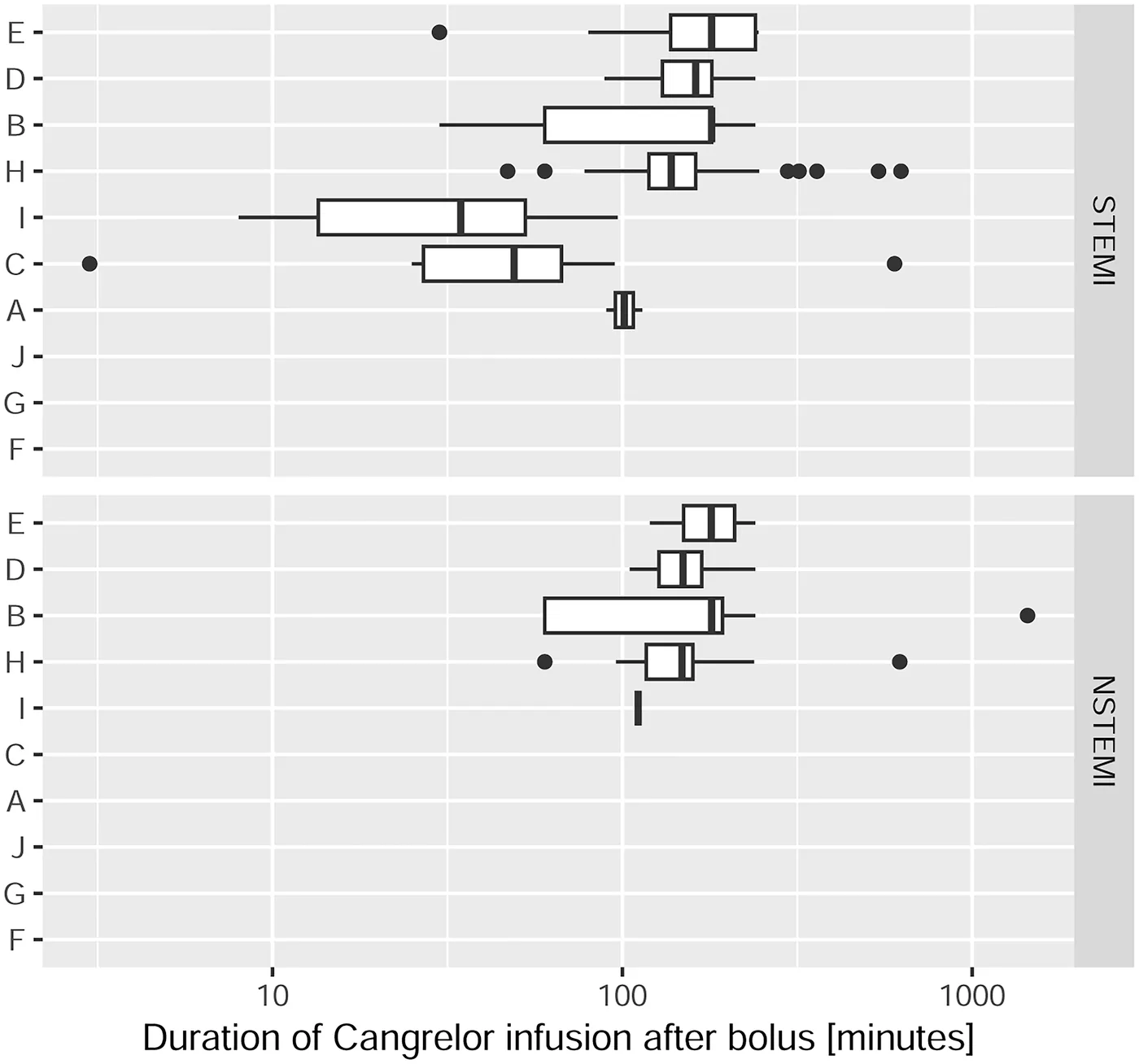
Cangrelor infusion duration by participating center. Box plots showing the duration of cangrelor infusion after bolus administration according to participating center, stratified by STEMI and NSTEMI. Boxes indicate the IQR, center lines the median, whiskers extend to 1.5   ×   IQR, and points represent outliers. The *x*-axis is shown on a logarithmic scale.

Cangrelor was administered for a median duration of 148 min (IQR 60), with substantial inter-center variability (Figure 1). The bolus and infusion rate were administered according to official recommendations across all centers.

Before cangrelor administration, 431 patients (68.8%) received heparin and aspirin only, while 195 patients (31.2%) received an additional oral P2Y₁₂ inhibitor: 64 patients (32.8%) received clopidogrel, 105 (53.8%) ticagrelor, and 26 (13.3%) prasugrel.

Transition to oral P2Y₁₂ inhibitors occurred with clopidogrel in 151 (25% of 602) patients, ticagrelor in 295 (49% of 602), and prasugrel in 156 (26% of 602), the rest (24, 3,8%) did not undergo oral transition. Administration prior to cangrelor discontinuation was observed in 6% of patients receiving clopidogrel, 27% receiving ticagrelor, and 4% receiving prasugrel, while the remainder received the oral P2Y₁₂ inhibitor at the time of cangrelor discontinuation or thereafter.

Among patients who received an oral P2Y₁₂ inhibitor before the index PCI, 33% (65/195) were switched to a different oral P2Y₁₂ inhibitor following cangrelor discontinuation. Switching occurred in 45% of patients initially treated with clopidogrel (14% to ticagrelor and 32% to prasugrel), 25% of those initially treated with ticagrelor (11% to clopidogrel and 14% to prasugrel), and 39% of those initially treated with prasugrel (23% to clopidogrel and 15% to ticagrelor).

The median interval between oral P2Y₁₂ inhibitor administration and cangrelor discontinuation was 15 min (IQR −10 to 35 min). Median pre-discontinuation administration times were 30 min for clopidogrel and 15 min for prasugrel among patients receiving the respective agents before cangrelor cessation.

In 107 patients (23%), the oral P2Y₁₂ inhibitor was administered concomitantly with cangrelor discontinuation, in 266 patients (57%) prior to discontinuation, and in 97 patients (21%) following termination of the infusion.

**Figure 2:**
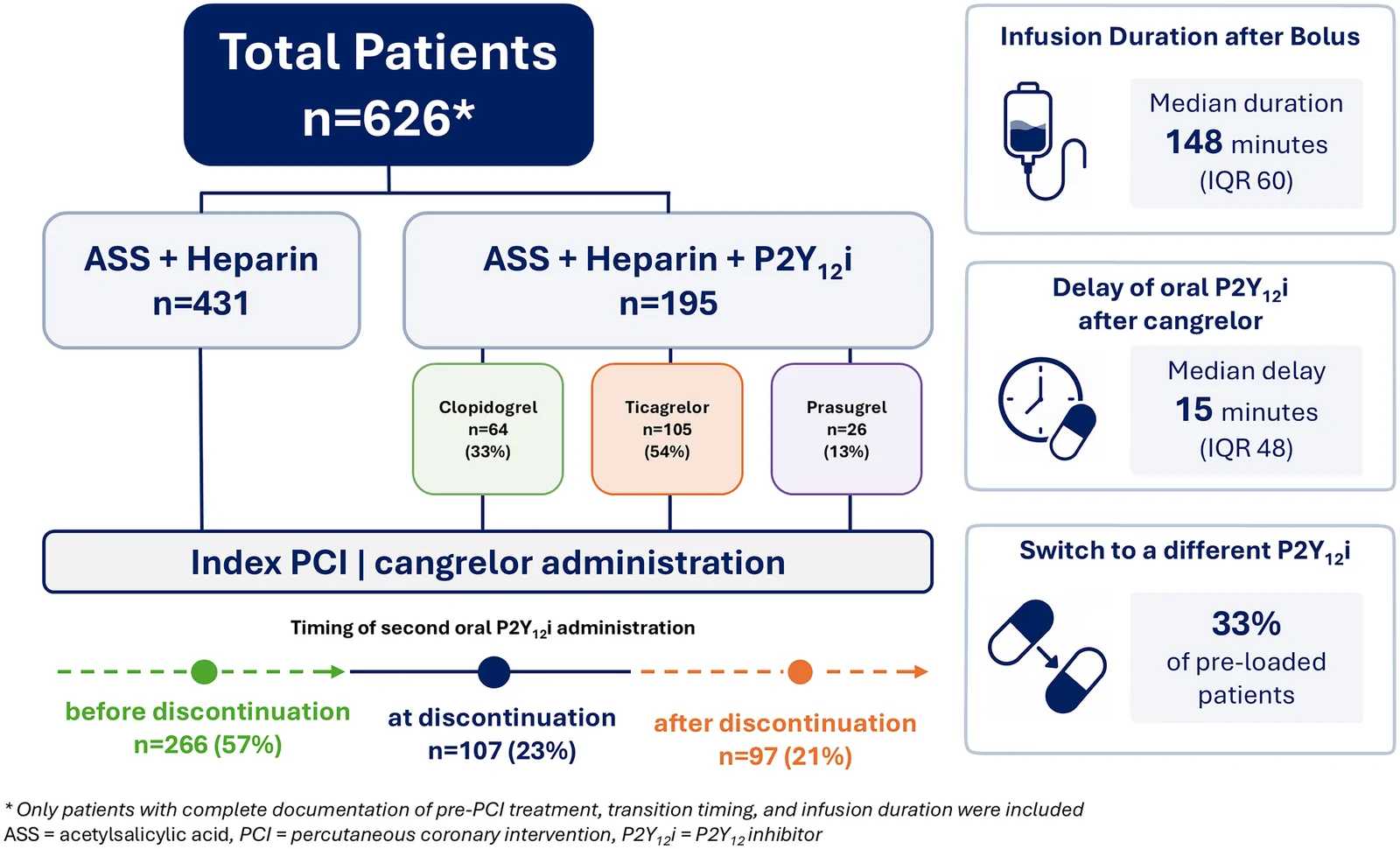
Timing of cangrelor administration and transition to oral P2Y12 inhibitor therapy: study flow of pre-PCI antiplatelet therapy, timing of cangrelor administration relative to discontinuation of oral P2Y12 inhibitor therapy, cangrelor infusion duration, and transition to oral P2Y12 inhibitor therapy. ASS, aspirin; APT, antiplatelet therapy; PCI, percutaneous coronary intervention; P2Y₁₂i = P2Y₁₂ inhibitor.

An overview of cangrelor administration is presented in Figure 2.

### 3.3 Clinical outcomes

During the index hospitalization, 85 patients (14%) experienced a major adverse cardiovascular event (MACE), while 46 patients (7%) developed major bleeding. Median cangrelor infusion duration was 139 vs. 142 min for patients with and without MACE, respectively (*p* = 0.134), and 131 vs. 122 min for patients with and without major bleeding, respectively (*p* = 0.256).

Among the 46 patients who experienced major bleeding, 13 (7%) received clopidogrel, 21 (7%) ticagrelor, and 12 (8%) prasugrel (*p* = 0.410). Differences in the choice and timing of oral P2Y12 inhibitor administration were observed across the study population. The incidence of MACE and major bleeding was similar across groups (*p* = 0.172 and *p* = 0.483, respectively).

Clinical outcomes are summarized in Table 2.

**Table 2:** Clinical outcomes.

	Total	
Items	n	m
Bleeding (≥BARC 3)	626	46 (7%)
BARC 3a		18 (3%)
BARC 3b		15 (2%)
BARC 3c		4 (<1%)
BARC 5a		7 (1%)
BARC 5b		2 (<1%)
MACE	626	85 (14%)
Cardiovascular death		62 (10%)
Stroke		0
Recurrent myocardial infarction		9 (1%)
Refractory angina		8 (1%)
Unplanned revascularization		6 (1%)

## 4 Discussion

This nationwide study provides real-world evidence on the clinical application of cangrelor, particularly its use alongside preprocedural oral P2Y_12_ inhibitors in patients with acute coronary syndrome (ACS). Substantial inter-center variability was noted in both the duration of cangrelor infusion and the timing of transition to oral P2Y_12_ inhibition following PCI. Approximately one-third of ACS patients received dual antiplatelet therapy (DAPT) with aspirin and an oral P2Y_12_ inhibitor prior to percutaneous coronary intervention (PCI) and the initiation of cangrelor.

Marked differences were observed among participating centers regarding the duration of cangrelor infusion. Compared with the CHAMPION PHOENIX trial (median 129 min, interquartile range 120–146 min), our results demonstrate a substantially wider range of infusion times, including instances where cangrelor was administered for less than the recommended minimum of 120 min (12, 13). This finding aligns with other real-world reports, such as the CAMEO registry, in which approximately 30% of patients received cangrelor infusions lasting under two hours (14). Similarly, significant variability was noted in the timing of transition to postprocedural oral P2Y_12_ inhibition, consistent with findings from other observational analyses (9, 14).

Although high platelet reactivity has been linked to adverse cardiovascular outcomes in previous studies (11, 15, 16), our cohort showed similar rates of MACE and bleeding complications across the observed durations of cangrelor infusion and timings of oral P2Y12 inhibitor loading. These findings reflect variability in periprocedural antiplatelet management in current clinical practice. The clinical significance of deviations from established guideline recommendations cannot be determined from these observational data.

Approximately one-third of ACS patients received pre-hospital dual antiplatelet therapy (DAPT) with aspirin and an oral P2Y_12_ inhibitor before the initiation of periprocedural cangrelor. Among the orally administered P2Y_12_ inhibitors given prior to PCI, ticagrelor was the most frequently used, followed by clopidogrel and prasugrel, consistent with observations from the Swedish Coronary Angiography and Angioplasty Registry, as well as its widespread adoption in contemporary clinical practice (8). However, in contrast to our cohort, almost all patients received ticagrelor and no use of prasugrel was reported (8).

Ticagrelor, in contrast to clopidogrel and prasugrel, does not exhibit a known pharmacodynamic interaction with Cangrelor (17). *In vitro* studies have demonstrated that clopidogrel and prasugrel compete with cangrelor for binding at the P2Y_12_ receptor, potentially diminishing their antiplatelet efficacy when administered simultaneously or in close temporal proximity (18, 19). Although current guidelines recommend cangrelor primarily for P2Y_12_ -inhibitor–naïve patients, real-world data, including our findings, indicate that it is occasionally administered to individuals already pre-treated with oral P2Y_12_ inhibitors (2, 8). However, evidence regarding the clinical outcomes of this combined antiplatelet approach remains limited (20).

Pretreated patients with potentially impaired oral P2Y_12_ inhibitor absorption (e.g., STEMI receiving morphine, cardiogenic shock, or endotracheal intubation) may represent the most justifiable off-label indication for cangrelor. In these patients, although oral loading may have been administered, gastrointestinal absorption is unpredictable, whereas cangrelor provides immediate and reliable platelet inhibition independent of gastrointestinal function. Nevertheless, the transition back to oral P2Y_12_ inhibitors remains susceptible to drug–drug interactions, and the clinical benefit of this strategy has not been established in randomized trials. While concomitant cangrelor may augment platelet inhibition in ticagrelor-pretreated patients during the infusion period, patients pretreated with thienopyridines are at risk of a transient gap in platelet inhibition if reloading occurs prematurely after discontinuation of cangrelor, once the active metabolite of the oral agent has been cleared. Reassuringly, adherence to this potentially harmful strategy was low across participating centers.

The decision to administer cangrelor despite prior oral P2Y₁₂ inhibitor loading is likely influenced by several clinical and procedural factors that were not systematically captured in the present registry. Delayed gastrointestinal absorption due to opioid administration, refractory angina, anticipated high thrombotic risk, or operator preference may all contribute to this treatment strategy. Likewise, angiographic characteristics such as impaired baseline TIMI flow, high thrombus burden, or no-reflow phenomena may have influenced the decision to initiate cangrelor but were not available for analysis. Consequently, the observed variability in cangrelor use should be interpreted within the context of these unmeasured factors. Prospective registries incorporating detailed procedural, angiographic, and pharmacological data are warranted to better understand the clinical rationale underlying different cangrelor administration strategies.

Several important limitations should be acknowledged when interpreting our findings. First, the non-randomized, registry-based design does not permit causal inference and is inherently vulnerable to residual confounding. While adjustments were made for relevant clinical and procedural variables, additional unmeasured patient-, lesion-, or procedure-related characteristics may have influenced the observed outcomes. Therefore, the findings should be interpreted as associative rather than causal. Second, treatment decisions, including cangrelor use and infusion duration, may have been influenced by local practice patterns and individual physician preferences. A further limitation is the seven-year enrolment period, during which changes in clinical practice, concomitant therapies, and standards of care may have occurred. Although these changes likely affected the entire cohort, the possibility of residual confounding due to temporal trends cannot be excluded.

## 5 Conclusion

This nationwide real-world study highlights considerable variability in the clinical use of Cangrelor and its integration with oral P2Y_12_ inhibitors.

## Data Availability

The raw data supporting the conclusions of this article will be made available by the authors, without undue reservation.
